# Effects of intrauterine growth restriction on embryonic hippocampal dentate gyrus neurogenesis and postnatal critical period of synaptic plasticity that govern learning and memory function

**DOI:** 10.3389/fnins.2023.1092357

**Published:** 2023-03-16

**Authors:** Camille M. Fung

**Affiliations:** Division of Neonatology, Department of Pediatrics, University of Utah School of Medicine, Salt Lake City, UT, United States

**Keywords:** intrauterine growth restriction, fetal growth restriction, hypertensive disease of pregnancy, embryonic neurogenesis, hippocampal dentate gyrus, learning and memory, Wnt signaling, critical period of synaptic plasticity

## Abstract

Intrauterine growth restriction (IUGR) complicates up to 10% of human pregnancies and is the second leading cause of perinatal morbidity and mortality after prematurity. The most common etiology of IUGR in developed countries is uteroplacental insufficiency (UPI). For survivors of IUGR pregnancies, long-term studies consistently show a fivefold increased risk for impaired cognition including learning and memory deficits. Among these, only a few human studies have highlighted sex differences with males and females having differing susceptibilities to different impairments. Moreover, it is well established from brain magnetic resonance imaging that IUGR affects both white and gray matter. The hippocampus, composed of the dentate gyrus (DG) and cornu ammonis (CA) subregions, is an important gray matter structure critical to learning and memory, and is particularly vulnerable to the chronic hypoxic-ischemic effects of UPI. Decreased hippocampal volume is a strong predictor for learning and memory deficits. Decreased neuron number and attenuated dendritic and axonal morphologies in both the DG and CA are additionally seen in animal models. What is largely unexplored is the prenatal changes that predispose an IUGR offspring to postnatal learning and memory deficits. This lack of knowledge will continue to hinder the design of future therapy to improve learning and memory. In this review, we will first present the clinical susceptibilities and human epidemiology data regarding the neurological sequelae after IUGR. We will follow with data generated using our laboratory’s mouse model of IUGR, that mimics the human IUGR phenotype, to dissect at the cellular and molecular alterations in embryonic hippocampal DG neurogenesis. We will lastly present a newer topic of postnatal neuron development, namely the critical period of synaptic plasticity that is crucial in achieving an excitatory/inhibitory balance in the developing brain. To our knowledge, these findings are the first to describe the prenatal changes that lead to an alteration in postnatal hippocampal excitatory/inhibitory imbalance, a mechanism that is now recognized to be a cause of neurocognitive/neuropsychiatric disorders in at-risk individuals. Studies are ongoing in our laboratory to elucidate additional mechanisms that underlie IUGR-induced learning and memory impairment and to design therapy aimed at ameliorating such impairment.

## Introduction

Intrauterine or fetal growth restriction (IUGR/FGR) is defined as failed fetal weight gain based on the fetus’ genetic potential which is determined by gestational age, race, and sex (Mandruzzato et al., 2008). There is a high incidence of IUGR in developing countries where macro- and micro-nutrient deprivation is the primary cause compared to the developed world where uteroplacental insufficiency (UPI) is the major cause. The etiologies of UPI are multiple, but the underlying theme of all centers around decreased uteroplacental blood flow to the fetus. Hypertensive disease of pregnancy (HDP) has become a prime factor for UPI as the incidence of metabolic syndrome increases in adults and in pregnant women (Cho et al., 2016; Grieger et al., 2018). IUGR occurs when maternal hypertension develops or worsens with chronic hypertension during pregnancy, decreasing uteroplacental blood flow and nutrient delivery to the fetus (Figueras and Gardosi, 2011). Alarmingly, maternal COVID-19 infection has recently been associated with a 1.6× increased odds of developing HDP (Rosenbloom et al., 2021; Marchand et al., 2022), adding to the growing problem of UPI.

## Neurological complications of IUGR

An offspring’s future neurological outcome is increasingly recognized to be associated with the prenatal environment. Specifically, suboptimal fetal growth is known to lead to motor and sensory neurodevelopmental deficits, cognitive and learning impairments, as well as cerebral palsy (Gagnon, 2003; Yanney and Marlow, 2004; Guellec et al., 2011; von Beckerath et al., 2013; Blair and Nelson, 2015; Gilchrist et al., 2018). Unfortunately, the inconsistent clinical definition of IUGR has probably underestimated the true prevalence of these neurologic sequelae (Fung and Zinkhan, 2021). An inaccurate distinction between a fetus who is constitutionally small-for-gestational (SGA) but is healthy compared to a fetus with poor weight gain due to a true pathological compromise of IUGR muddles the literature. Traditionally, the diagnosis of IUGR solely referred to an infant with a birth weight of <10th percentile or >2 standard deviations below the mean for gestational age and sex (Kingdom and Smith, 2000). Moderate or severe IUGR was defined as birth weight of 3 −10% or <3%, respectively. More recently, the ability to incorporate indices of UPI especially with umbilical artery Doppler flow velocimetry has attempted to distinguish offspring with true IUGR from those who are constitutionally small (Society for Maternal-Fetal Medicine, Martins et al., 2020). It is our opinion that future studies relating to IUGR must continue to distinguish between the two in order to identify offspring who are at the highest risk for future neurologic compromise.

### IUGR alters brain blood flow distribution

As mentioned, UPI is the greatest contributor to IUGR in high-income countries (Figueras and Gratacos, 2014). UPI causes chronic hypoxemia, reduces nutrient availability and utilization by the fetus, culminating in fetal growth deceleration. The growth-restricted fetus adapts by blood flow re-distribution to favor essential organs such as the brain, heart, and adrenal glands (Poudel et al., 2015). However, this cardiac output re-distribution to protect brain growth, previously termed asymmetric growth restriction or “brain sparing,” does not protect the fetus from potential abnormal brain development (McMillen et al., 2001; Poudel et al., 2015). Recent studies in IUGR fetuses showed that reduced middle cerebral artery (MCA) Doppler pulsatility index (PI), commonly used to assess severity of UPI, may only detect an *advanced* stage of blood flow re-distribution. This is because anterior cerebral artery vasodilates before MCA vasodilates to shunt blood flow to the frontal region which is the earliest compensatory response in face of hypoxemia (Figueroa-Diesel et al., 2007; Rossi et al., 2011). As chronic hypoxemia ensues, changes in MCA perfusion favors the basal ganglia at the expense of the frontal region, and reduced MCA PI becomes detectable (Hernandez-Andrade et al., 2012).

### IUGR alters brain architecture

Regardless of the success of brain blood flow re-distribution, the neuropathological consequences of IUGR on the developing brain are often heterogeneous and unpredictable (Fung and Zinkhan, 2021). Multiple factors govern the eventual neurological phenotype to include the timing and severity of the *in utero* compromise, whether the infant is born at preterm or term, and whether other co-existing complications impact the brain’s developmental trajectory (Miller et al., 2016). In general, two broad factors appear to be critical to neurodevelopmental outcome: the severity of the placental dysfunction coupled with the gestational age at onset plus the gestational age at delivery (Baschat, 2011).

Intrauterine growth restriction causes widespread deficits in brain structure that includes reduction of both white and gray matter of the cortex, hippocampus, and cerebellum. This leads to reduced head circumference as volume is reduced throughout the brain (Fung and Zinkhan, 2021). In fact, small head size during infancy is a strong predictor for poor neurodevelopmental outcome (Gale et al., 2006; Ochiai et al., 2008). Reduced myelin content, delayed myelination, and/or reduced connectivity have all been shown to contribute to white matter volume reduction. Diffusion tensor imaging (DTI) assessment of fractional anisotropy (FA) *via* magnetic resonance imaging is a useful tool in neonatal medicine to provide detailed microstructural organization and integrity of the white matter tracts without ionizing radiation to infants. Normally in healthy, myelinated white matter, FA values are high and radial diffusivity is low, demonstrating that water molecules preferentially diffuse in the direction of fiber tracts (Miller et al., 2016). DTI imaging in 12 month-old IUGR infants has shown altered FA in the large white matter tracts of the corpus callosum and internal capsule (Padilla et al., 2014). Additionally, reduced global and local network efficiency using graph model measures as well as reduced connectivity in long-range cortico-basal ganglia connections in the prefrontal and limbic networks in preterm infants born with IUGR were seen in whole brain connectome analyses (Batalle et al., 2012; Fischi-Gomez et al., 2015).

Experimental models of IUGR in rodents, guinea pigs, rabbits, and sheep have highlighted significant developmental gray matter alterations that contribute to brain injury. In particular, gray matter volume reduction was attributed to a total reduction in neural cell number, altered synaptic formation, and/or cell migration defects resulting in simplified gyrification (Miller et al., 2016). Similar to human IUGR brain imaging, reduced volume of motor and visual cortices, hippocampus, basal ganglia, and cerebellum contributed to the morphological gray matter alterations in IUGR animal models (Mallard et al., 1998; Rees and Harding, 2004; Lodygensky et al., 2008). Volume loss was partly explained by neuronal loss across a number of brain regions including the hippocampus (Fung et al., 2012; Miller et al., 2014), and surviving neurons showed selective changes in the morphology of the hippocampal neuronal dendrites (Dieni and Rees, 2003). Moreover, axonal injury and nerve conduction defects were caused by developmental dysmaturation of oligodendrocytes and delayed myelination of axons (Olivier et al., 2007; Chang et al., 2018). Overall, there appears to be an imbalance in the regulation of many neuronal and oligodendroglial processes that affect proliferation, differentiation, and apoptosis within the developing brain of growth-restricted offspring (Fung and Zinkhan, 2021).

### IUGR alters brain function

Given the aforementioned white and gray matter changes, it follows then that IUGR would significantly affect brain function. Depending on the brain regions involved, these sequelae can also have varied manifestations. One study cites that infants with IUGR were 4−6 times more likely to develop cerebral palsy than infants without IUGR (Jarvis et al., 2003). A systematic review of early childhood neurodevelopmental outcomes after IUGR reviewed 731 studies of which 16 were included (Levine et al., 2015). Of note, only eight studies included abnormal Doppler parameters in their IUGR definition. Amongst these, eleven studies found poorer neurodevelopmental outcomes while ten studies found motor delays, eight studies found cognitive delays, and seven studies found language delays. Other delays included social development, attention, and adaptive behavior (Levine et al., 2015).

For those studies that incorporated Doppler flow parameters, absent or reversed end diastolic velocity in the umbilical artery confers the highest risk for neurologic deficits (Fung and Zinkhan, 2021). School-aged children with severe IUGR were found to perform worse on assessment tasks for cognition, motor function, behavior, and educational achievements than children who only had mild to moderate IUGR (Schreuder et al., 2002). Because UPI often leads to preterm birth, some investigators note that preterm birth may exacerbate the neurologic impact of IUGR and being born small-for-gestational age (Yanney and Marlow, 2004). Indeed, a large prospective French study examined the neurological outcomes of school-aged children who were born with appropriate weight versus small-for-gestational age at 24−28 weeks or 29−32 weeks. They showed that the 24−28 weeks cohort regardless of their birth weight had similar cognitive deficits, inattention-hyperactivity, and school difficulties to those infants who were small-for-gestational age at 29−32 weeks. The appropriately grown 29−32 weeks fared better than the small-for-gestational 29−32 weeks group but still had mild cognitive and behavioral issues (Guellec et al., 2011). In addition to the IUGR severity, gestational age, and birth weight, the child’s sex also contributes to differential outcomes. For example, 24−29 weeks male preterm infants with early-onset IUGR had poorer school achievement and behavioral problems when compared to 24−29 weeks preterm female or appropriately grown preterm male infants (Parkinson et al., 1981; Morsing et al., 2011). Streimish et al. (2012) additionally found that in female infants, more severe growth restriction was a strong predictor for impaired cognition whereas less severe IUGR was not. Depression, schizophrenia, and mood disorders are other adverse outcomes associated with IUGR (Fung and Zinkhan, 2021). As children grow into early adulthood, a study that followed IUGR term infants through 16−26 years of age in England showed that they had relatively lower academic achievement test scores, lower teacher rating of school success, and were less likely to have professional employment (Strauss, 2000). Therefore, it appears that brain changes in fetal life significantly impact multiple functional domains of the IUGR individual.

At this juncture, I will highlight two large prospective studies recently published in Norway and Finland that inspected the specific associations between maternal HDP with offspring neurological outcomes (Lahti-Pulkkinen et al., 2020; Sun et al., 2020). Lahti-Pulkkinen et al. (2020) found that HDP encompassing the entire spectrum of disease ranging from maternal gestational hypertension, chronic hypertension, preeclampsia and its severity, increased the offspring’s hazard of *any* childhood mental, psychological, and behavioral/emotional disorders at ages 6.4–10.8 years. Moreover, maternal HDP, diabetes mellitus, and overweight/obesity additively increased the offspring hazard of these mental disorders (Lahti-Pulkkinen et al., 2020). Contrary to prior studies, preterm birth only partially mediated the effects of any mental disorder in this study (Lahti-Pulkkinen et al., 2020). The second retrospective study by Sun et al. (2020) examined the associations between preeclampsia in *term* pregnancies and neurologic outcomes for a mean follow up of 14 years. Children exposed to preeclampsia were at increased risks for attention-deficit-hyperactivity disorder, autism spectrum disorder, epilepsy, intellectual disability, and cerebral palsy (Sun et al., 2020). These two studies are important to consider because they provide evidence that pregnancy complications such as HDP have become an important contributing factor to poor childhood/adulthood neurologic outcomes independent of premature birth.

## Animal models of IUGR used as tools to interrogate mechanisms of IUGR-induced hippocampal injury and associated learning and memory deficits

**FIGURE 1:**
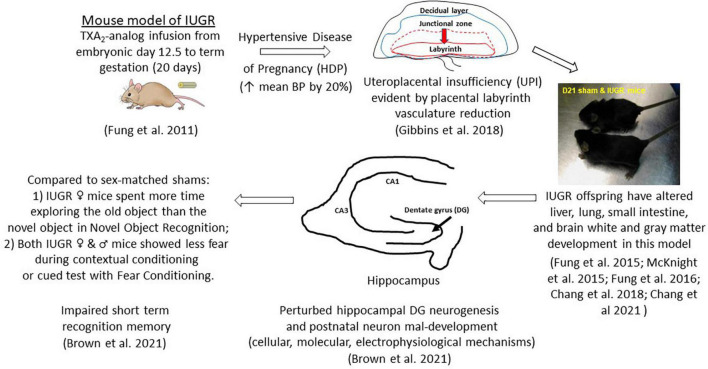
Uteroplacental insufficiency (UPI) originating from hypertensive disease of pregnancy (HDP) affects fetal hippocampal dentate gyrus neurogenesis in IUGR offspring which leads to postnatal neuron mal-development and learning and memory deficits. Other organ systems are also affected in this mouse model of IUGR.

Many animal models of IUGR exist to subserve different experimental paradigms. They range from nutritional models of caloric restriction, low protein restriction, iron deficiency, and over-nourishment of adolescent pregnant sheep, to surgical and hypoxic models that include bilateral or single uterine ligation, uteroplacental embolization, carunclectomy, hypoxic chambers, and maternal glucocorticoid administration or stress model performed in guinea pigs, rabbits, rodents, or sheep (Vuguin, 2007; Miller et al., 2016). Each model bears its strengths and weaknesses; but because the most common reason for IUGR in the developed world is HDP as previously mentioned, our laboratory saw a need to devise a novel translationally relevant model mimicking the pathophysiology of human HDP to dissect at mechanisms of IUGR-induced learning and memory deficits (Figure 1). We chose the laboratory mouse over other animal species in order to utilize the more advanced transgenic technology and neurobehavioral paradigms established in mice. In addition, ample literature exists to describe the normal embryonic and postnatal hippocampal development in rodents particularly relating to neural circuitry within the hippocampus.

It is important at this point to review the rodent hippocampal neural circuitry in order to understand its role in learning and memory function. The mature hippocampus is comprised of four CA fields, the DG, and the subiculum (Agster and Burwell, 2013). Information flows throughout the hippocampus *via* three important pathways. The major afferent input is the perforant pathway whereby axons from layer II of the entorhinal cortex project into the DG and CA3 (Gilchrist et al., 2018). DG granule cell axons, known as mossy fibers, then project into CA3 pyramidal cells (Gilchrist et al., 2018). The CA3 pyramidal cell axons then project into CA1 *via* Schaffer collaterals (Gilchrist et al., 2018). CA1 axons exit back to entorhinal cortex *via* the subiculum creating a unidirectional circuit for memory creation. In adult humans and animals, the generation and survival of newly formed DG granule cells in the subgranular zone (SGZ), termed adult neurogenesis, contributes to ongoing hippocampal-dependent learning and memory (Gilchrist et al., 2018). In fact, the relationship between adult neurogenesis and learning and memory abilities is reciprocal, meaning that hippocampal-dependent learning tasks increase the number of newly generated granule cells in the DG SGZ throughout the life of the organism (Gould et al., 1999). Embryonic neurogenesis follows the same neural circuitry as the adult. But instead of neurogenesis from the SGZ, neurogenesis begins in the ventricular zone of the cortical hem, which is situated in the dorsal medial region of the telencephalon, and gives rise to choroid plexus, Cajal-Retzius cells, and the hippocampus (Moore and Iulianella, 2021).

It follows then that an understanding of the timeline of human hippocampal development should provide an insight into the vulnerability of the developing hippocampus to IUGR injury. The unfolded hippocampus arises by 13−14 weeks of human gestation and reaches its adult shape by 25 weeks (Kier et al., 1997). As such, disruptions during the second trimester will likely impose a profound effect on hippocampal development given that neurogenesis is active. Of interest, HDP typically presents after 20 weeks of gestation (Mammaro et al., 2009), creating an environment of UPI and decreasing fetal growth over time. It is important to point out though that hippocampal development follows a protracted time course that begins *in utero* but continues after birth. Therefore once the IUGR insult is removed at delivery, compensatory mechanisms can attempt to right the wrong in the proper setting. In fact, the hippocampus undergoes a growth spurt in the first 2 years of life, continuing to grow linearly until early adolescence when it reaches the adult size (Uematsu et al., 2012). Of importance, the DG and CA subregions continue to develop into early childhood versus the subiculum which is fairly mature at birth. The DG-CA3 connection is the last to mature in the hippocampus, contributing to adult-like memory formation as well as most prone to ongoing developmental aberrancies. During early childhood, the hippocampus and the cortical regions such as the prefrontal cortex and superior frontal gyrus become increasingly integrated to perform memory encoding, retention, and retrieval processes (Riggins et al., 2016). Paradoxically, the hippocampus also progressively segregates from brain regions unrelated to memory function, a basis for improvement in age-related episodic memory function.

### Mouse model of HDP using thromboxane A_2_-analog micro-osmotic pump infusion to replicate UPI

The creation of this model is to replicate HDP as the etiology of UPI to target the sequence of events occurring in embryonic neurogenesis. Because mothers with hypertension overly produce the potent vasoconstrictor, thromboxane A_2_ (TXA_2_), over the vasodilator, prostacyclin (Liu et al., 1998; Shah and Khalil, 2015), we implant a micro-osmotic pump containing either a vehicle (sham surgery) or a TXA_2_-analog, U-46619, to pregnant mouse dams beginning at embryonic day (E) 12.5 until term gestation of 20 days in C57Bl/6J mice (Figure 1; Fung et al., 2011). The rationale behind inducing IUGR at E12.5 in mice is multiple: (1) embryonic DG neurogenesis is just beginning; (2) the layered structure of the definitive murine placenta is established to support the ongoing pregnancy; and (3) the placental labyrinth layer, which is the maternofetal exchange layer, undergoes tremendous fetal vasculature expansion both in size and in complexity from E12.5 until delivery (Simmons et al., 2008). We have shown that our IUGR placentae have substantial reduction in CD31 (PECAM-1) abundance beginning at E17.5 and E19 denoting reduced fetal vasculature and replicating UPI as the etiology of IUGR (Figure 1; Gibbins et al., 2018). Moreover, our placental study also found that at early-to-mid gestation (E15.5−17.5), hypertensive placentae mounted compensatory mechanisms to preserve fetal growth by increasing placental efficiencies (defined as grams of fetus produced per gram of placenta) and maintaining abundance of important nutrient transporters such as glucose transporters and neutral amino acid transporters (Gibbins et al., 2018). Interestingly, they also increased fatty acid transporter four and fatty acid translocase expression to augment fatty acid accretion in the fetus (Gibbins et al., 2018). As the placental vascular network diminished over late gestation (E19), placental efficiency diminished, and fetal growth failed. Hypertensive placentae in late gestation also exhibited a sex-differential expression of neutral amino acid and fatty acid transporters despite showing fetal growth failure in both sexes (Gibbins et al., 2018).

After birth, our IUGR pups weighed 15% less and had lighter brain, lung, liver, and kidney weights but had similar nose-to-anus lengths compared to sham pups (Fung et al., 2011). IUGR offspring remained growth restricted from birth through postnatal day (P) 21 (Figure 1; Fung et al., 2011). IUGR male pups caught up to sham males in weight by P28, whereas IUGR female pups caught up by P77 (Fung et al., 2011). IUGR males surpassed sham males in weight by P238 with IUGR females weighing similar to sham females throughout the first year of life (Fung et al., 2011). This differential growth trajectory is important to highlight as we consider the neurological phenotype in this model in the following sections. But before we move on to the characterization of the IUGR hippocampal DG neurogenesis and neurobehavioral phenotype, it is worth mentioning that other organs such as the liver, small intestine, and brain white matter are also impacted by IUGR in this model (Fung et al., 2015, 2016; McKnight et al., 2015; Chang et al., 2018, 2021).

Armed with our mouse model of IUGR, we examined the cellular, molecular, and neurobehavioral phenotypes to understand the prenatal and postnatal changes induced by IUGR.

## Effects of IUGR on hippocampal-based learning and memory function using our mouse model of HDP

Memory is classified as implicit or explicit. Implicit memory refers to non-conscious learning that is evident through performance and does not require access to any conscious memory contents (Schacter et al., 1998). Explicit memory, on the other hand, involves encoding, storage, and retrieval that is recalled with conscious effort and is highly plastic, permitting the creation of new associations (Kandel et al., 2000). We interrogated learning and memory function on adult sham and IUGR pups using novel object recognition (NOR) as our test of implicit memory and fear conditioning for explicit memory. NOR relies on exposing mice to two objects on the first training day. On the second day, one old object is substituted with a novel object. The natural tendency for mice is to explore the novel object if they remembered seeing the old object. Fear conditioning involves two parts: contextual conditioning and cue test after a training day. Mice are first placed into a chamber on day 1. In contextual conditioning, an audible tone is first presented followed by a brief foot shock. With learning, mice will associate hearing the tone with freezing behavior as they anticipate a foot shock after the tone. In cued test, mice are placed into the same chamber where the foot shock was delivered on the previous day. Mice that do not freeze in either scenario have not learned the associations (Rodriguiz and Wetsel, 2006).

We discovered that IUGR male and female offspring showed impaired short term recognition memory *via* NOR and fear conditioning (Brown et al., 2021). Specifically, compared to sex-matched offspring, IUGR females explored the old object longer than the novel object after a day of training in NOR. IUGR males, on the other hand, preferentially explored the novel object as expected (Figure 1; Brown et al., 2021). Moreover, both IUGR females and males spent less time freezing or showed less fear during contextual conditioning and cued test when compared to their sex-matched shams (Brown et al., 2021). Therefore, we have validated that our mouse model of IUGR indeed had learning and memory deficits and would provide a model to decipher prenatal events that predispose IUGR offspring to future learning and memory deficits.

## Effects of IUGR on embryonic DG neurogenesis using our mouse model of HDP

**FIGURE 2:**
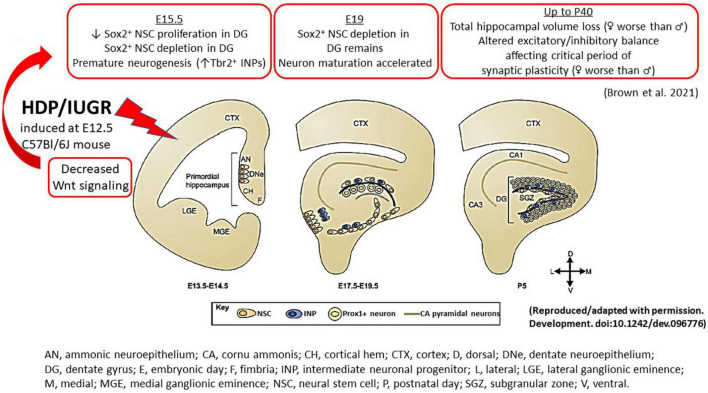
An illustration summarizing the hippocampal findings in our mouse model of IUGR. During normal embryonic HDG development, neural stem cells give rise to intermediate neuronal progenitors, which migrate tangentially and eventually give rise to Prox1^+^ granule neurons. In the postnatal brain, NSCs reside in the subgranular zone and continue to generate Prox1^+^ granule neurons. The cortical hem, a transient structure formed during development, is the source of a number of patterning morphogens, such as Wnt and BMP proteins, which play critical roles in cell fate specification during hippocampal neurogenesis. In our mouse model of HDP, IUGR was induced at E12.5. At E15.5 (3 days after maternal hypertension), IUGR hippocampus showed decreased canonical and non-canonical Wnt signaling which is accompanied by decreased Sox2^+^ NSC proliferation and depletion. By contrast, IUGR promoted premature neuronal differentiation by increasing % Tbr2^+^ INPs. At E19, % Sox2^+^ NSCs remained diminished but neuron maturation proceeded in an unhindered fashion to generate more NeuroD^+^ neuronal progenitors and Prox1^+^ granule neurons in IUGR. In postnatal life up to day 40, IUGR hippocampi had total volume loss. Novel to the field, IUGR perturbed the critical period of synaptic plasticity to create an excitatory/inhibitory imbalance. AN, ammonic neuroepithelium; CA, cornu ammonis; CH, cortical hem; CTX, cortex; D, dorsal; DNe, dentate neuroepithelium; DG, dentate gyrus; E, embryonic day; F, fimbria; INP, intermediate neuronal progenitor; L, lateral; LGE, lateral ganglionic eminence; M, medial; MGE, medial ganglionic eminence; NSC, neural stem cell; P, postnatal day; SGZ, subgranular zone; V, ventral.

The prenatal events that set an IUGR offspring up for future learning and memory deficits have remained elusive thus far partly due to an inability to study fetal human brain tissues longitudinally. The use of animal models thus has become a powerful tool to track cellular and molecular alterations that may underlie IUGR-induced brain injury. Using our mouse model, we have shown that at E15.5, 3 days after maternal hypertension in pregnancy, IUGR offspring of both sexes had fewer % Sox2^+^ neural stem cells (NSCs) in the ventricular and marginal zones in hippocampal DG anlage (Figure 2; Brown et al., 2021). Additionally, fewer % EdU^+^ proliferative cells delivered as a 2 h pulse label were noted in the ventricular zone with fewer % phosphohistone H3 (pHH3) at serine 10 co-staining, showing fewer proliferative cells completed mitosis within the cell cycle.

Interestingly, instead of proliferation, IUGR DG anlage of both sexes was noted to have increased % Tbr2^+^ intermediate neuronal progenitors (INPs) at E15.5, showing *premature* neuronal differentiation compared to age-matched sham pups (Figure 2; Brown et al., 2021). As gestation progressed to E19, a day prior to delivery, % Sox2^+^ NSCs remained diminished but proliferation *via* EdU pulse labeling was restored (Brown et al., 2021). The % Tbr2^+^ INPs, NeuroD^+^ neuronal progenitors, and Prox1^+^ granule neurons were increased in E19 IUGR dentate anlage compared to age-matched sham pups showing that once neuronal differentiation proceeded, neuronal maturation occurred in an unhindered fashion in both sexes (Figure 2; Brown et al., 2021).

To our knowledge, this is the first description of IUGR-induced NSC depletion and premature neurogenesis in the developing hippocampal DG (Brown et al., 2021). A recent study examined the effects of IUGR on fetal cortical neurogenesis in a murine maternal hypoxia-induced IUGR model and found a significant reduction in the thickness of the cerebral cortical which is accounted for by decreases in layer-specific neurons at birth (Chou et al., 2022). They noted that a significantly lower number of Pax6^+^/EdU^+^ fetal NSCs in the IUGR group after 48 h of EdU incorporation, suggesting decreased self-renewal of fetal NSCs after IUGR (Chou et al., 2022) similar to our hippocampal findings. They also found a skew in cell fate decision with an accelerated transitioning from a stem to a progenitor state, resulting in a decrease in stem cell pool size (Chou et al., 2022). Unlike our data, however, they found significantly more early-born Ctip2^+^/EdU^+^ labeled neurons in cortical plate but fewer late-born Satb2^+^/EdU^+^ neurons in the intermediate zone or Brn2^+^/EdU^+^ neurons in the ventricular zone, suggesting a delayed transitioning of fetal NSCs from producing early-born to late-born neurons (Chou et al., 2022). They noted a delay in cell cycle progression, particularly in the G2/M phase during inward interkinetic nuclear migration, as responsible for the delayed transitioning of fetal NSCs from producing early-born to late-born neurons (Chou et al., 2022). This is different from our developing DG findings in which neuronal maturation occurred without any hindrance in IUGR and the neuronal populations remain more numerous than their sham counterparts throughout gestation.

To investigate the molecular pathways that may be responsible for our early cellular alterations, RNA-sequencing was used to detect the differential transcript expression between sham and IUGR mouse hippocampi at E15.5. Of the 16,873 mouse genes analyzed, 611 protein-coding gene transcripts were differentially expressed (Brown et al., 2021). ∼70% of the IUGR-affected genes were found to have downregulated expression. Ingenuity Pathway Analysis identified the canonical (Wnt/β-catenin) and non-canonical Wnt (planar cell polarity = PCP) signaling pathways as predicted to have downregulated activity in E15.5 IUGR hippocampi (Brown et al., 2021). The glutamate receptor signaling pathway, which shares the same differentially expressed genes as the endocannabinoid neuronal synapse pathway, cAMP response element-binding protein (CRBE) signaling, neuropathic pain signaling pathway, synaptic long-term potentiation, and synaptogenesis signaling pathway, was predicted to have upregulated activity (Brown et al., 2021).

Knowing the important role of stem and progenitor cell maintenance with Wnt signaling (Zhou et al., 2006), we believe that a loss of Wnt signaling in IUGR could be the first inciting event that causes NSC depletion and premature embryonic neurogenesis. Both the canonical and non-canonical Wnt pathways require Wnt ligand binding to their cognate receptor types such as the Frizzled proteins, but the two downstream pathways diverge substantially after ligand binding (Brown et al., 2021). Our RNA-seq data identified several Wnt-related genes that are worth discussing. Wnt3a is notable here not only because of its greatest decreased expression in IUGR but it also marks the cortical hem from which induction and patterning of the hippocampus originates beginning at E 9.75 in mice (Roelink and Nusse, 1991; Grove et al., 1998). In Wnt-3a mouse mutants, medial hippocampal fields are absent and lateral hippocampal fields are severely reduced because of the lack of proliferative expansion of the caudomedial stem and progenitor cells (Roelink, 2000). In the case of IUGR, Wnt3a reduction could lead to the reduction of Sox2^+^ and EdU^+^ NSCs because of decreased proliferation. Additional support lending to the relevance of Wnt signaling in IUGR is decreased Dkk1 and Wif1 expression found in our model, which encode Wnt pathway inhibitors. These genes would be expected to be downregulated to allow residual Wnt activity to work without devastating detriment to the developing hippocampus evident by the decreased Sp5 expression, often used as a Wnt pathway readout.

Consistent with our observation of decreased NSC proliferation, we postulate that premature cell cycle exit would drive progenitors in the IUGR brain to differentiate toward the neuronal lineage, indicated by a significant increase in Tbr2^+^ INPs. Previous work by Hodge et al. showed that the transcription factor Tbr2 is critically essential for DG neurogenesis in both developing and adult mice (Hodge et al., 2012). In the absence of Tbr2, INPs are depleted despite augmented NSC proliferation. They also found that Tbr2 is enriched at T-box binding sites in the Sox2 locus to repress Sox2 expression, suggesting that Tbr2 may promote the commitment of pluripotent NSCs to neuronal-specified INPs through this mechanism. Once the transition to Tbr2-expressing INPs begins, neuronal maturation proceeds in IUGR to express other maturing neuronal transcription factors such as NeuroD and Prox1 to complete embryonic hippocampal DG neurogenesis (Seki et al., 2014). Studies are ongoing to validate this postulate in cell cycle exit by defining the phases of cell cycle *via* flow cytometry in Sox2^+^ and Tbr2^+^ populations of the sham and IUGR hippocampi.

## Effects of IUGR on postnatal hippocampal DG neuronal development using our mouse model of HDP

### Hippocampal volumes

Given that the murine hippocampus completes its development by ∼P30 (Seki et al., 2014), we investigated hippocampal volumes at P18 and P40 to track the developmental progression of this structure in postnatal life. Consistent with human literature and other animal models of IUGR, we found decreased hippocampal volumes at both ages our IUGR mice (Figure 2; Brown et al., 2021). When males and females were separated, IUGR females had the smallest volumes compared to IUGR males and shams. The mechanisms behind this sex difference are currently unknown but intriguing, especially when we review the postnatal growth trajectories between the two sexes.

In our model, all pups are cross-fostered by unmanipulated mouse dams made pregnant at the same time as the dams destined for sham or IUGR surgery. Once all pups are born, they are nursed by these unmanipulated dams to avoid any lactation issues associated with anesthesia and micro-osmotic pump implantation. Even though we have never measured milk volumes consumed by pups, we assume that sham and IUGR pups are receiving similar amounts of dam milk during postnatal rearing. With “normal” nutrition, our IUGR females achieve catch-up growth 7 weeks later than IUGR males and they never surpass sham females’ weights over the first year of life. There is ample literature to show that attaining catch-up growth is paramount to determining future neurologic health. Adequate catch-up reduces learning and memory impairment amongst other neuropsychological detriments (Geva et al., 2006; Fattal-Valevski et al., 2009; Duran Fernandez-Feijoo et al., 2017). The lack of catch-up growth in IUGR females could incur an additional postnatal growth restriction which will impact brain growth and function. At this juncture, the reasons behind why IUGR females fail to attain catch-up growth as compared to IUGR males are unknown. We believe that one contributor may relate to the sexually-dimorphic placental nutrient transporter expression found in late gestation as previously discussed. Placentae that supported IUGR males upregulated neutral amino acid transporters as well as fatty acid transporters as compensatory mechanisms to improve fetal nutrient accretion (Gibbins et al., 2018). It follows then that IUGR males may also be at increased risk for earlier onset metabolic syndrome with faster catch-up growth, supported by their heavier body weights at 8.5 months compared to sham males. The competing interests of attaining catch-up growth to improve neurocognitive and neuropsychiatric outcomes but to avoid worse metabolic outcomes remain a clinical challenge in the care of these IUGR infants.

As mentioned earlier in this article, postnatal changes to hippocampal neurons and their dendritic and axonal connections in experimental animal models of IUGR are well described (see reviews Miller et al., 2016; Gilchrist et al., 2018). For example, IUGR guinea pig neonates have fewer CA1 pyramidal cells (Mallard et al., 2000), which is consistent with reduced CA1 pyramidal layer in IUGR juvenile rats (Camprubi Camprubi et al., 2017). Reduction of the CA1 stratum oriens width was seen in fetal guinea pigs with IUGR showing decreased dendritic and axonal growth (Mallard et al., 1999). Sheep models of IUGR have also shown that the DG width is reduced in IUGR (Rees et al., 1988), and alterations to the DG granule cell morphology have been found with reduced dendritic outgrowth in guinea pigs with IUGR (Dieni and Rees, 2003). Our laboratory has taken a newer angle in the postnatal characterization by examining the critical period of synaptic plasticity.

### Critical period of synaptic plasticity

**FIGURE 3:**
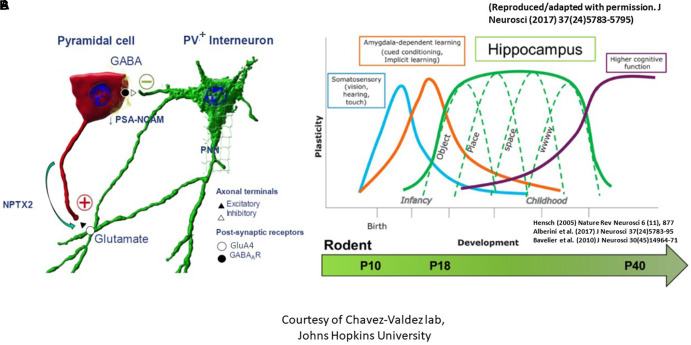
The critical period of synaptic plasticity. **(A)** It is governed by an interaction between the excitatory CA pyramidal cell (denoted as red) and the inhibitory parvalbumin^+^ (PV^+^) interneuron (denoted as green). Opening of the critical period is governed by decreased polysialylation of the neural cell adhesion molecule (PSA-NCAM) in the excitatory pyramidal cell neuronal membranes as well as advanced maturation of PV^+^ interneurons with increased number of glutamate decarboxylase synaptic boutons/axon terminals to result in GABAergic network development. Consolidation of the critical period, on the other hand, is marked by increased neuronal pentraxin 2 (NPTX2) production by CA pyramidal cells, which later localizes to the GluA4 subunit of AMPA receptors on the postsynaptic PV^+^ interneurons. Lastly, closure of the critical period is marked by advanced myelination and formation of perineural nets (PNNs) which support NPTX2’s proximity to PV^+^ interneurons. **(B)** Regional critical periods of synaptic plasticity progress sequentially in brain development, beginning with the somatosensory cortex, amygdala, then the hippocampus. Postnatal ages at which this sequential maturation of the critical period in normal rodent brain development is delineated at the bottom [adapted from Alberini and Travaglia (2017) and Blair et al. (2010)].

Since the classical experiments performed by Hubel and Wiesel in the visual cortex in the 1960s (Hubel and Wiesel, 1962; Wiesel and Hubel, 1963), it has been well accepted that a critical period exists when neurons are particularly susceptible to modification by experience, which is concurrent with large-scale anatomical changes that become irreversible after the closure of this critical period (Hensch, 2004, 2005; St Pierre et al., 2022). The classical critical period of plasticity occurs mostly in juvenile animals. It has been considered as a central mechanism for establishing fine-tuned neuronal circuits in the developing brain characterized by profound refinement and remodeling of brain circuits with extensive neurite outgrowth, synaptic formation, and pruning to create mature networks shaped by experience (Hensch, 2004, 2005). The critical period in the dorsal hippocampal CA subregions, marked by boundaries that are determined by complex biochemical and structural events between the excitatory pyramidal cells and the inhibitory parvalbumin^+^ (PV^+^) interneurons, strives to create an excitatory to inhibitory balance (St Pierre et al., 2022). The *opening* of this critical period is governed by decreased polysialylation of the neural cell adhesion molecule (PSA-NCAM) in the excitatory pyramidal neuronal membranes as well as advanced maturation of PV^+^ interneurons with increased number of glutamate decarboxylase (GAD) synaptic boutons/axon terminals to result in GABAergic network development (Figure 3A; Lisman et al., 2012; Travaglia et al., 2016a,b). *Consolidation* of this critical period, on the other hand, is marked by increased neuronal pentraxin 2 (NPTX2 or Narp) production by CA pyramidal cells, which later localizes to the GluA4 subunit of AMPA receptors on the postsynaptic PV^+^ interneurons. Lastly, advanced myelination and formation of perineural nets (PNNs) which support NPTX2’s proximity to PV^+^ interneurons *close* the critical period (Makinodan et al., 2012; Xiao et al., 2017). It is worth mentioning that regional critical periods of synaptic plasticity progress sequentially in brain development, beginning with the somatosensory cortex, amygdala, then the hippocampus (Figure 3B). Temporal progression of the rodent hippocampal critical period is postulated to start between P17-P21 and to close during late adolescence after P30 (Carstens et al., 2016; Gao et al., 2018). Dysregulation of the timing of the critical period perturbs the excitatory/inhibitory balance and is postulated to contribute to developmental disorders and psychopathology in at-risk individuals (Aceti et al., 2015; Meredith, 2015).

In our mouse model, we examined these phases of the critical period at P10, P18, and P40. We found that at P10, no difference was seen in the number or volume of either PV^+^ interneurons or GAD 65/67^+^ puncta between sham and IUGR (St Pierre et al., 2022). At P18, however, despite no difference in PV^+^ interneurons, we found increased GAD 65/67^+^ puncta and volume in IUGR mice in CA pyramidal cell layer compared to sham, suggesting an advanced GABAergic development in IUGR. At P40, this difference disappeared in IUGR as the sham group caught up in GAD^+^ bouton development (St Pierre et al., 2022). Concurrently at P18, we saw decreased PSA-NCAM expression in the pyramidal cell membranes in IUGR, which normally acts to limit the formation of perisomatic inhibitory GABAergic synapses, but in this case has permitted the accelerated GABAergic synaptic formation in IUGR mice. Following the development of inhibitory inputs into pyramidal cells, NPTX2 is then normally upregulated to allow excitatory synapse formation. Contrary to our prediction, IUGR mice had decreased NPTX2^+^ puncta in the CA subregion particularly in CA3 (St Pierre et al., 2022). At P40, NPTX2^+^ puncta deficits remained but were attenuated than P18. Collectively, this shows that IUGR has stunted excitatory synapse formation during consolidation. Finally, closure of the critical period is marked by oligodendrocyte maturation and myelination. For the first time, we noted a sex-specific difference in that IUGR female mice only had decreased length, surface area, and volume of myelinated axons as denoted by myelin basic protein (MBP) staining at P40 (St Pierre et al., 2022). In fact, MBP^+^ axon length directly correlated with NPTX2 volume in the CA radiatum layer. The number of pyramidal neurons was similar between sham and IUGR therefore neuronal dropout was not the reason for decreased myelination. Additionally, we examined PNN formation which is needed to stabilize NPTX2^+^ excitatory synapses onto PV^+^ interneurons. Despite no differences in PNN number between P40 sham and IUGR mice, PNN volumes and densities were decreased in CA subregions of IUGR female mice (St Pierre et al., 2022). Within all of the aforementioned changes, CA3 displayed more constant changes than CA1 affirming the importance of DG-CA3 mossy fiber pathway in establishing learning and memory function. In summary, our discovery of the disturbance in all phases of the critical period especially in IUGR females may additionally support worse learning and memory deficits seen in this sex. Studies are underway to examine further on oligodendrocyte maturation defect as it relates to glial dysregulation.

## Conclusion

One in seven delivery hospitalizations in 2019 was related to HDP according to the Centers for Disease Control and Prevention.^1^ The increased prevalence for HDP includes primigravida, advanced maternal age, obesity, and diabetes, all of which have increased in the U.S. in recent years. It follows then that the prevalence of IUGR is expected to be on the rise and the short- and long-term complications that impact almost every organ will continue remain a public health problem. These maternal risks occur independently of maternal COVID-19 infection which is also recently recognized to be an additional risk factor for HDP development and COVID-19 infection will likely become an endemic problem worldwide. It is now clear that both the white and gray matter of the developing brain are equally vulnerable to IUGR brain injury, and that such a prenatal injury impacts multiple brain regions and neural processes critical for later executive functions. Our laboratory is dedicated to following the longitudinal prenatal and postnatal alterations of embryonic and adult hippocampal neurogenesis and neuronal development as an underlying basis for learning and memory deficits. We are also interested in following the sex-specific differences behind the varied susceptibilities between IUGR male and female offspring. Certainly, other studies utilizing other animal or *in vitro* models that examine other brain regions with their mechanistic discoveries will permit a thorough understanding of the impact IUGR imposes on our children. As clinicians who take care of pregnant mothers and their offspring or scientists who have a vested interest in understanding the pathogenetic mechanisms of IUGR, we must continue to pursue studies and seek research funding to uncover the neural changes behind IUGR-induced neurocognitive and neuropsychiatric conditions if we are to improve our children’s future mental health.

## Author contributions

CF performed the literature search of the current state of science to be discussed in the review, wrote up the entire review, and selected or created figures to be included in the manuscript.

## Footnotes

1
https://www.cdc.gov/media/releases/2022/p0428-pregnancy-hypertension.html
